# The impact of temperature on microbial diversity and AOA activity in the Tengchong Geothermal Field, China

**DOI:** 10.1038/srep17056

**Published:** 2015-11-26

**Authors:** Haizhou Li, Qunhui Yang, Jian Li, Hang Gao, Ping Li, Huaiyang Zhou

**Affiliations:** 1 School of Life Sciences and Technology, Tongji University, Shanghai 200092, China; 2 State Key Laboratory of Marine Geology, School of Ocean and Earth Science, Tongji University, Shanghai 200092, China; 3 School of Engineering, Anhui Agricultural University, Hefei 230000, China.

## Abstract

Using a culture-independent method that combines CARD-FISH, qPCR and 16S rDNA, we investigated the abundance, community structure and diversity of microbes along a steep thermal gradient (50–90 °C) in the Tengchong Geothermal Field. We found that Bacteria and Archaea abundance changed markedly with temperature changes and that the number of cells was lowest at high temperatures (90.8 °C). Under low-temperature conditions (52.3–74.6 °C), the microbial communities were dominated by Bacteria, which accounted for 60–80% of the total number of cells. At 74.6 °C, Archaea were dominant, and at 90.8 °C, they accounted for more than 90% of the total number of cells. Additionally, the microbial communities at high temperatures (74.6–90.8 °C) were substantially simpler than those at the low-temperature sites. Only a few genera (e.g., bacterial *Caldisericum*, *Thermotoga* and *Thermoanaerobacter,* archaeal *Vulcanisaeta* and *Hyperthermus*) often dominated in high-temperature environments. Additionally, a positive correlation between Ammonia-Oxidizing Archaea (AOA) activity and temperature was detected. AOA activity increased from 17 to 52 pmol of NO_2_^−^ per cell d^−1^ with a temperature change from 50 to 70 °C.

Microorganisms are diverse and abundant in geothermal fields on Earth^1^. Some microbes are able to survive at temperatures as high as 122 °C, challenging our understanding of the physical and chemical constraints on life^2^. Understanding how living communities survive and are structured under various environmental conditions in geothermal regions is important because geothermal fields are similar to the postulated early chemical environment on Earth. Recently, research has focused on the physicochemical parameters, such as temperature, pH and nutrient supply, that shape microbial diversity, activity and community structure in geothermal fields. For example, physicochemical conditions have been shown to exert strong control over microbial ecology and distribution^3^. In addition, Ammonia-Oxidizing Archaea (AOA) may play important roles in carbon and nitrogen cycles in these hyperthermal regions^4^,^5^,^6^. Ammonium oxidation as an ancient form of energy conservation is consistent with the postulated chemically driven nitrogen cycle of early Earth^7^.

The Tengchong Geothermal Field (24 °57′N, 98 °26′E) is the largest and most active geothermal region in Asia^8^,^9^,^10^. This park sits along arched fault structures, which are similar to those in Yellowstone National Park and submarine volcano regions. In this area, there are numerous hot springs, pools, fumaroles, geysers and hydrothermal explosions with temperatures ranging from 50 to 97 °C and pH ranging from 1.8 to 9.3^11^,^12^,^13^. The wide diversity of physical and chemical environmental factors provides a multitude of niches for extremophile microorganisms in microbial communities^14^,^15^. Recently, several studies have focused on microbial cultivation, identification and taxonomy in this region. Hedlund *et al.*^16^ isolated diverse members of the Aquificales from geothermal springs in Tengchong, including *Hydrogenobacter*, *Hydrogenobaculum*, and *Sulfurihydrogenibium.* Hou *et al.*^10^ found that the bacterial phylum Aquificae and the archaeal phylum Crenarchaeota were dominant in Tengchong hot springs. Briggs and colleagues^17^ revealed seasonal variation in the structure of microbial communities in Tengchong hot springs. Wang *et al.*^18^ found that temporal variation in geochemistry can alter microbial community structure and diversity. In addition, Jiang and co-workers^19^ reported that AOA were active in samples of Tengchong hot springs with temperatures as high as 94 °C, and Zhang *et al.*^20^ examined the relative abundances of the amoA gene and glycerol dialkyl glycerol tetraether lipids (GDGTs), which are potential biomarkers specific to AOA, in Tengchong hot springs. However, very little is known about microbial abundance and the contribution of AOA to nitrification in Tengchong.

In this study, a culture-independent approach that combined CARD-FISH, qPCR and a 16S rRNA gene library was used to investigate the abundance, community structure, and diversity of microbes along a steep thermal gradient in the Tengchong Geothermal Field, where the temperature conditions are the most variable across the soil profile. We also analysed the impact of temperature on the activity of AOA.

## Results

### Microbial distribution

**Figure 1:**
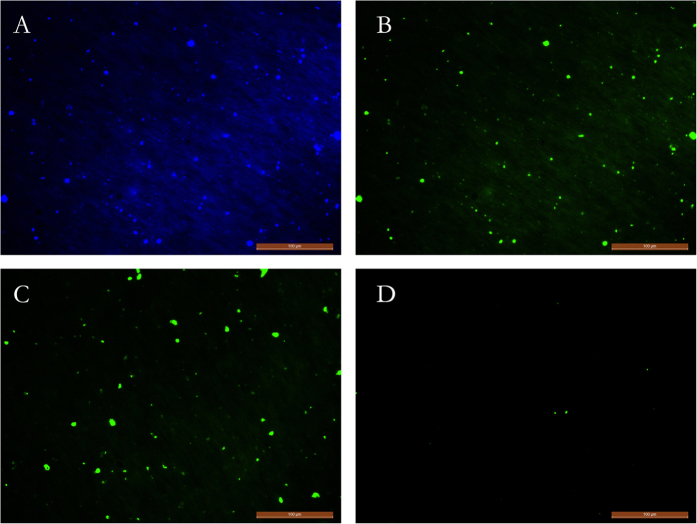
Photomicrographs of CARD-FISH stained samples. (**A**) DAPI stained. (**B**) Probe EUB338 targeted most of Bacteria. (**C**) Probe Arch958 targeted most of Archaea. (**D**) Probe Cren512 targeted most of AOA. Magnification = 200×, scale bar = 100 μm.

In this study, we used CARD-FISH to investigate the microbial distribution of samples of different temperatures. At 2 to 5 ng/μL, the probe EUB338 (Table 1, Fig. 1B, Fig. S1) mainly targeted Bacteria, whereas the probe Arch 915 (Fig. 1C) primarily targeted Archaea, and the probe Cren512 (Fig. 1D) mostly targeted AOA^21^. The average sum of Archaea plus Bacteria cell numbers detected by 16S rDNA fluorescent probes accounted for 60–95% of the total DAPI-stained cell numbers.

As shown in Table 2, total cell numbers decreased as the soil temperature increased, with 7.3 × 10^7^ cells g^−1^ soil at the lowest temperature (soil surface) and 4.0 × 10^3^ cells g^−1^ soil at the highest temperature (deepest layer). The low-temperature area had at least a 10^4^-fold greater number of cells than did the high-temperature area.

Similarly, the Bacteria and Archaea cell numbers also decreased from 6.6 × 10^7^ to 0.5 × 10^2^ cells g^−1^ soil and from 7.2 × 10^6^ to 3.6 × 10^3^ cells g^−1^ soil, respectively, as the soil temperature increased. However, the AOA numbers increased from 2.0 × 10^2^ to 1.1 × 10^3^ cells g^−1^ soil as the soil temperature increased.

Interestingly, at low temperatures (52.3–74.6 °C), the microbial communities were dominated by Bacteria, which contributed approximately 60–80% of the total number of cells. However, at 74.6 °C, Archaea became the dominant population, and at the highest temperature of 90.8 °C, it accounted for more than 90% of the total number of cells. 16S rDNA and amoA (encoding a subunit of the key enzyme ammonia monooxygenase) gene copies were quantified by real-time PCR (Table 2). We used the gene copy numbers to indirectly infer the microorganism distribution in this hyperthermophile environment. The microbial communities at different temperatures were largely consistent with the results from the CARD-FISH. The 16S rDNA gene copies of Bacteria and Archaea ranged from 8.78 × 10^7^ to 1.17 × 10^2^ copies g^−1^ soil and from 8.20 × 10^6^ to 3.89 × 10^3^ copies g^−1^, respectively. The amoA gene copies indicated that Archaeal ammonia oxidizers were more abundant in soils than their Bacterial counterparts, and the abundance of AOA amoA copies increased to 0.8–34.7% Archaea as the soil temperature increased.

### Microbial diversity

**Figure 2:**
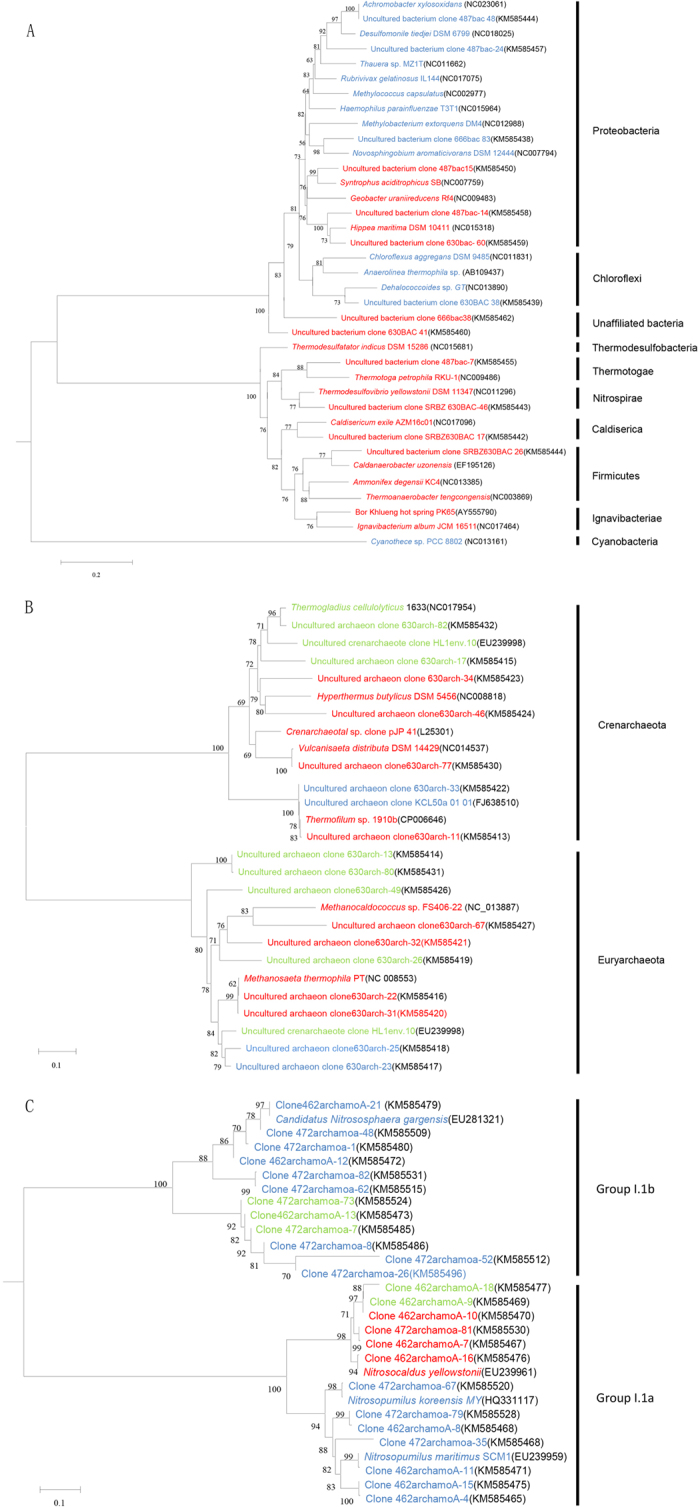
Maximum likelihood phylogenetic tree from Bacteria (A), Archaea (B) and AOA (C). Bootstrap (1,000 replicates) values of >50 are indicated at the nodes. The scale bar represents the estimated sequence divergence. Color code: red = high- temperature (74.6–90.8 °C), blue = low-temperature (52.3–74.6 °C), green = all samples (52. 3–90.8 °C).

In our study, we chose one specific site to collect our samples. These samples presented a narrow range of geochemical conditions within a wide range of temperatures across a 50-cm soil depth (Table 3). The temperature conditions were most variable across the soil profile. A clone library was used to quantitatively assess the effect of temperature on microbial diversity. During sampling, temperature was considered as a main factor of interest, and five sites were selected for sampling (52.3 °C, 64.3 °C, 74.6 °C, 80.2 °C, 90.8 °C). The number of clones in each sample represented 76 to 100% coverage for the clone libraries. OTUs were defined as groups of sequences differing by 3% (16S rDNA) and 2% (amoA) at the DNA level. Many of the detected species were candidate species with no cultured representatives. In each soil sample, approximately 30–100 microorganisms with high-quality 16S rDNA and amoA sequences were obtained. The phylogenetic distribution of the microbes found in the Tengchong Geothermal Region is shown by the tree in Fig. 2. A detailed inventory of the microorganismal OTUs detected in each 16S rDNA clone library is given in Table S1.

**Figure 3:**
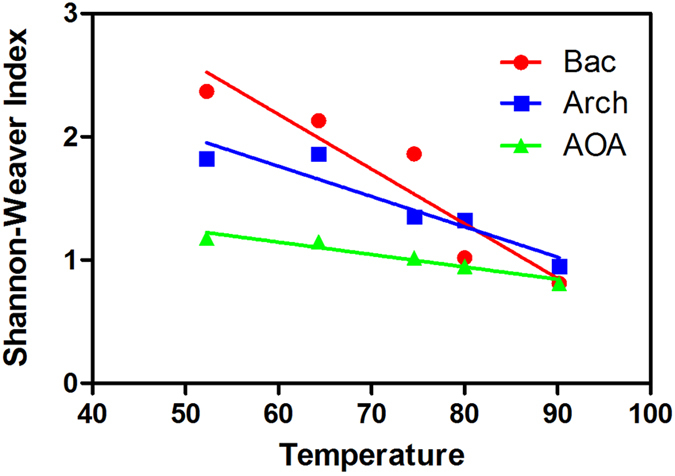
Temperature versus Shannon-Weaver index for each site in the current study. For this hot spring, an inverse linear trend (dotted line) between T and the Shannon–Weaver index is evident.

A very obvious change in soil community diversity was apparent across the thermal gradient (Fig. 3). The microorganismal community profiles at the hottest soil layer were substantially simpler than those of the lower temperature sites. Peak diversity for all samples was observed at 52.3 °C (Shannon–Weaver index: Bacteria, 2.89; Archaea, 1.87), with lower diversity under hotter conditions (Fig. 3). The bacterial diversity analysis revealed that the community in this thermal region was dominated by five phyla: Proteobacteria, Firmicutes, Nitrospirae, Thermotogae and Cyanobacteria. The greatest diversity was observed in the phylum Proteobacteria, with eleven genera present belonging to the classes Alphaproteobacteria, Betaproteobacteria and Gammaproteobacteria (Table S1).

**Figure 4:**
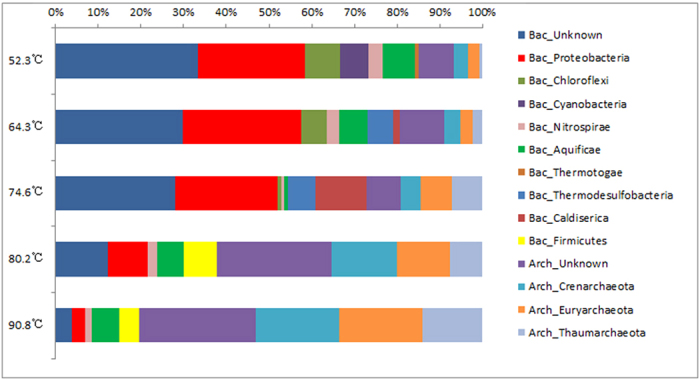
The microbial communities in different temperature soil layers.

In addition, the distribution of phyla varied with temperature. Proteobacteria (5–30%, Fig. 4) was represented at both low and high temperatures, but its relative abundance was higher at low temperatures (52.3–74.6 °C). Firmicutes appeared at high temperatures (74.6–90.8 °C) but not at low temperatures (52.3–74.6 °C). For these samples, an inverse linear trend (dotted line) between T and the Shannon-Weaver index is evident (Fig. 3).

The genera *Hippea*, *Syntrophus* and *Geobacter* in the phylum Proteobacteria were more adapted to hyperthermal environments, whereas the genera *Methylobacterium, Novosphingobium, Achromobacter*, *Desulfomonile*, *Rubrivivax, Haemophilus*, *Sorangium* and *Thauera* were only detected at low temperatures.

Members of the phylum Firmicutes reported in thermal environments, such as *Ammonifex*, *Caldanaerobacter* and *Thermoanaerobacter*, were only present in hyperthermal regions (>90 °C), where the soil temperature conditions were similar to the optimal growth temperatures of these genera.

We further constructed Archaeal 16S rDNA gene clone libraries. The analysis indicated a high biodiversity within the Bacterial clone library and a much lower diversity within the Archaeal clone library. The Archaea community was dominated by the phyla Euryarchaeota and Crenarchaeota in all conditions (50–90 °C), comprising 32 ± 16% and 45 ± 23% of the total Archaea, respectively. Under relatively high temperature conditions (74.6–90.8 °C), the Archaeal community was less diverse than in lower-temperature habitats (Table 3). In high temperature samples, the Shannon-Weaver index was 0.92, and in low temperature samples, the index was 1.85 (Table 3).

In the soil samples, we found that the phylum Crenarchaeotal component consisted primarily of the genera *Vulcanisaeta, Thermogladius*, *Thermofilum* and *Hyperthermus.* The phylum Euryarchaeota component consisted primarily of the genera *Methanosaeta* and *Methanocaldococcus.* However, some sequences were associated with an unknown genus. The genera *Vulcanisaeta*, *Thermofilum*, *Hyperthermus*, *Methanocaldococcus* and *Methanosaeta* were dominant in hyperthermal environments.

For amoA gene diversity in this geothermal region, we only amplified Archaeal amoA gene fragments. However, no AOB amoA genes were detected. Under the high temperature conditions, the dominant OTUs were close to *Nitrosocaldus*, whereas under low temperatures the dominant OTUs were closest to *Candidatus Nitrososphaera*.

### Culture and activity of ammonia-oxidizing cells in the enrichment media

**Figure 5:**
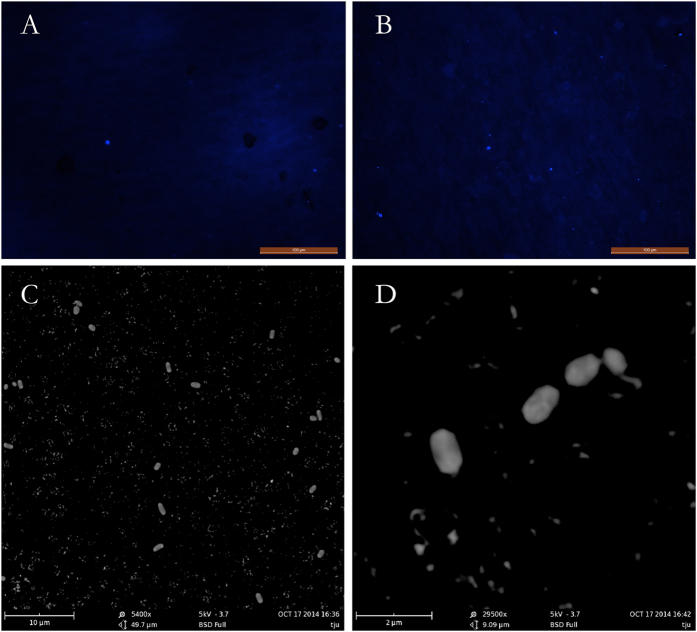
Photomicrograph of ammonia-oxidizing microbes in enrichment media (A), (B) Fluorescence image of cells stained with DAPI. Scale bar represents 100 μm **(C), (D)** Scanning electron micrograph of cells. Scale bar represents 10 and 2 μm, respectively.

**Figure 6:**
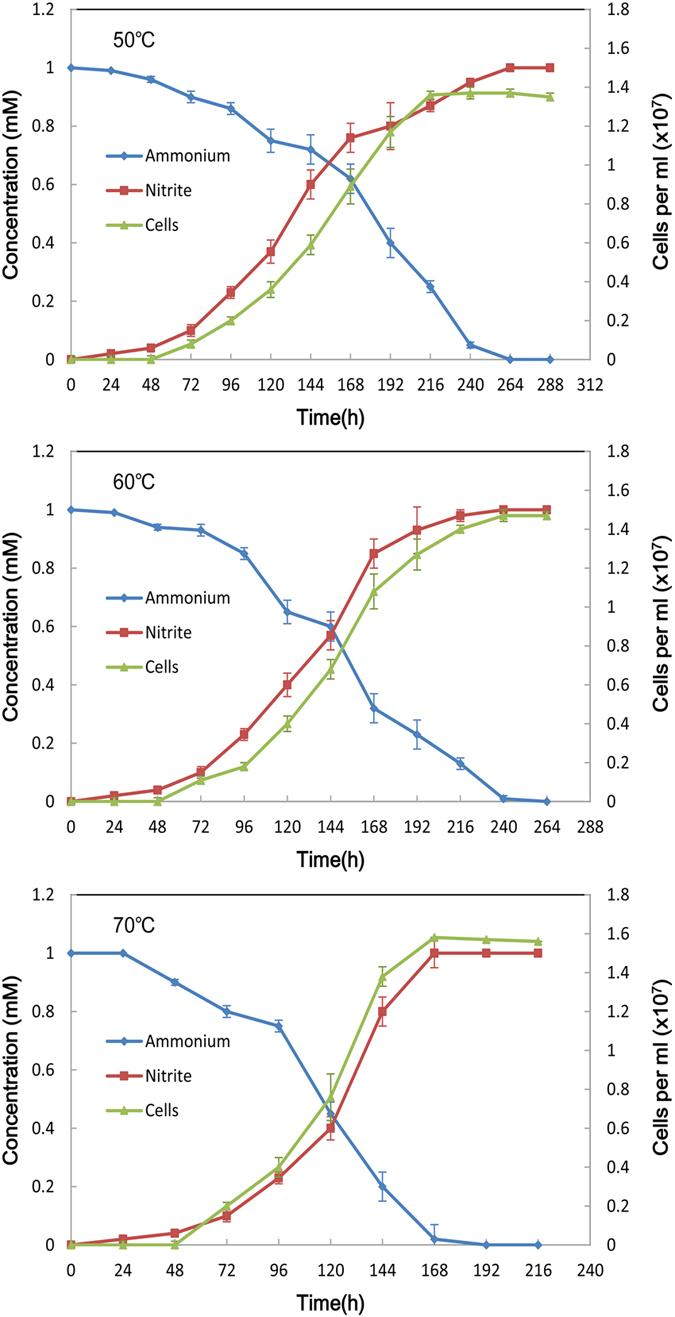
Activity of ammonia-oxidizing microorganisms in medium with different temperatures.

We set five temperatures (50, 60, 70, 80 and 90 °C) to cultivate ammonia-oxidizing microorganisms for 15–20 days under dark aerobic conditions. Microscopic examination revealed the microorganism populations present after cultivation in enrichment media (Fig. 5A,B). The ammonia-oxidizing activity of the microorganisms was determined based on nitrite production. All experiments were performed in triplicates and the reported values are average values from triplicates. Nitrite production was not observed in uninoculated media or at temperatures higher than 80 °C. The maximum cell numbers ranged from 1.35 × 10^7^ to 1.56 × 10^7^/mL across the different temperatures (Fig. 6). The oxidation rates of ammonia to nitrite corresponded with an increasing abundance of cells. Calculations of ammonia oxidation activity were based on the reported total number of cells and nitrite production in enrichment media^22^. The different temperature had somewhat influence on cell growth and activity. At a low temperature (50 °C), the ammonia oxidizing activity was low, at 17 pmol of NO_2_^−^ per cell per d. By contrast, at a higher temperature (70 °C), the activity was the highest observed, generating 52 pmol of NO_2_^−^ per cell per d (Fig. 6).

Electron photomicrographs (Fig. 5C,D) showed that most cells were small rods with a diameter of 0.55–0.93 μm and a length of 0.65–1.89 μm, and no flagella were observed.

## Discussion

Quantification of microbial populations is important for understanding many aspects of microbial ecology. CARD-FISH and real-time PCR were first described over a decade ago and were hailed as a breakthrough for microbial ecology^23^, and they are well known as effective techniques for detecting specific genes in environmental samples^24^. In the present study, these methods were used to determine the abundance of microorganisms in soil samples of different temperatures. The geothermal activity at the Tengchong Geothermal Field offers a unique opportunity to examine the impacts of environmental temperature on a naturally occurring soil microbial community. This study presented a narrow range of geochemistry conditions and a wide range of temperature values, and the results showed a dramatic shift in microbial diversity with changes in temperature. Temperature is likely a strong factor influencing microbial community structure. This interpretation is supported by the results of CARD-FISH and qPCR (Fig. 1, Table 2). The numbers of microorganisms were highest in the relatively low-temperature samples. The microorganism numbers correlated negatively with temperature. It is possible that the low-temperature environment provided some growth factors that are essential for microbial cells. The surface soil layer, which had a low temperature (approximately 50 °C), was dominated by Bacteria. At 74.6 °C, the Archaea dominated, and at the highest temperature (90.2 °C), more than 90% of the total number of cells were Archaea. This pattern suggests a high-temperature ecosystem, in which Archaea contributes a major fraction of the biomass. Archaea might adapt to high temperatures using a variety of strategies. Additionally, our data provide evidence for a high abundance of AOA in hot soils. The abundance of AOA increased slightly as temperature increased. The abundance of the AOA amoA gene was higher in hot soil than at low temperatures. These results indicate that AOA can adapt to a wide gradient in environmental temperature (50–90 °C) and are in agreement with previous studies carried out in the Great Basin and in Yellowstone National Park (United States) and in Kamchatka (Russia)^4^,^20^,^25^,^26^. Jiang *et al.*^19^ showed that the amoA gene of ammonia-oxidizing Archaea in Tengchong survived at temperatures between 74 °C and 94 °C, suggesting that Archaeal nitrification can potentially occur at near-boiling temperatures. Furthermore, ammonia is a common feature in many geothermal areas, and it is always permeated by the hydrothermal steam from the deeper soil. In our study, AOA were more abundant in deeper soil layers with high temperatures.

Analysis of 16S rDNA and amoA genes was used to characterize the taxa among samples. A significant correlation was observed between microorganism diversity and temperature (Fig. 3, Table 3). The environment had been selected by these thermophilic species in the high-temperature area.

Microbial communities in high-temperature environments are often dominated by a few types of microorganisms, and are often significantly less diverse than those in lower temperature habitats. Benson also reported that microbial diversity in the Hawaii Volcanoes National Park decreased as temperature increased^27^.

Furthermore, with increasing depth and temperature, the environment tends to become anaerobic. Oxygen concentrations in water are low at high temperatures, obeying Henry’s law. Most microbes are anaerobic under high-temperature conditions. For example, the Bacteria genera *Caldisericum, Syntrophus*, *Thermotoga* and *Thermoanaerobacter,* and the Archaea genus *Methanosaeta* are anaerobic microbes. A terminal RFLP examination of vertically distributed bacterial populations in rice paddy cores also showed significant changes in species composition along an oxygen gradient^28^. However, although AOA require oxygen for metabolism, we detected AOA at high temperatures. We speculate that there are two aspects that lead to this result. First, temperature may be the most important factor affecting AOA activity, even if the oxygen level is low. An increased temperature might accelerate the metabolism of microorganisms, resulting in increased AOA numbers. Second, previous research indicated that autotrophic ammonia/ammonium oxidation was restricted to aerobic ammonia-oxidizing bacteria (AOB). However, the discovery of anaerobic ammonia-oxidizing (Anammox) bacteria invalidated this idea. Peng *et al.*^29^ reported that AOA have been detected at high abundance in most of the global ocean, including environments such as pelagic oxygen minimum zones. The AOA can survive in both oxic and anoxic environments. It is possible that some unknown AOA could survive at high temperatures in a low-oxygen environment.

Meanwhile, microbial communities in high temperature areas were dominated by a few Archaea species, and the data suggest that these species are highly adapted to the geochemical and hydrological properties of this environment. We found that increasing temperature caused a decrease in the diversity of soil microbial communities (Table 3). Compared to previous studies of microbial diversity at the Tengchong Geothermal Field, our 16S rDNA sequences contained several novel lineages within the current phylum Crenarchaeota and within Candidatus members of the phyla Thaumarchaeota and Euryarchaeota. Overall, Tengchong microbial community structure was less diverse than that observed at Yellowstone National Park^30^,^31^, which is likely due to the significantly higher temperatures of Tengchong. Furthermore, we determined the amounts of AOA in thermal environments, which contributed to our understanding of the microbial ecosystem in thermophilic conditions.

AOA have been detected at sites in marine water columns of 2000 and 2956 m depth, at temperatures as low as 0.2 °C and at temperatures as high as 97.1 °C in volcano mud^25^,^32^,^33^,^34^,^35^, and they play a central role in the global nitrogen cycle^4^. Up to 4 × 10^11^ kg of N cycles through the ocean each year^36^, and almost all of this N must be nitrified at least once^37^. Despite recent advances in our understanding of nitrogen cycling activities in soils, fresh and marine waters, and sediments^7^,^25^,^26^,^38^, knowledge gaps in high temperature ecosystems have been slow to fill^20^.

In this study, we determined the impact of temperature on the activity of ammonia-oxidizing microorganisms. We focused on geothermal environment microorganisms, including enriched and characterized ammonia-oxidizing microorganisms from Tengchong geothermal soil. We found that these microorganisms could survive under enrichment at temperatures as high as 70 °C. The different temperature had somewhat influence on cell growth and activity. Our results indicated that the ammonia-oxidizing activity is slightly higher at 70 °C (52 pmol of NO_2_^−^ per cell per d) than that of 17 pmol of NO_2_^−^ per cell per d at a lower temperature (50 °C) (Fig. 6), the ammonium oxidation rates seem similar across depth and temperature. De La Torre *et al.*^39^ have cultivated a thermophilic Archaea (‘*Candidatus Nitrosocaldus yellowstonii*’ belongs to Crenarchaeota) at up to 74 °C showing ammonia-oxidizing properties. Based on our detected microorganisms (Fig. 2), Crenarchaeota were also found to be abundant in Tengchong soil samples by amoA clone libraries. Therefore, Crenarchaeota could potentially be one source of microorganisms being responsible for ammonium oxidation in our study. At same time, our data are consistent with Hamilton *et al.*^40^, showing that Archaea crenarchaeol can be abundant in organisms thriving at high temperatures. Our enrichment method enabled us to study the characteristics of thermal AOA, a potentially environmentally significant group of organisms in the submarine volcano region nitrogen cycle. This study increases our understanding of how microbial community structure and AOA activity vary across a wide range of environmental temperatures and how microorganisms contribute to biogeochemical cycles in thermal environments.

Ammonium oxidation as an ancient form of energy conservation is consistent with the postulated chemically driven nitrogen cycle of early Earth, as it allows for the formation of ammonia at high temperatures. Importantly, the research presented here represents a survey of microorganisms distributed across high-temperature habitats in the Tengchong Geothermal Field, which are similar to the postulated chemical environments of early Earth.

## Materials and Methods

### Sample collection

The study was conducted in experimental fields located at a Tengchong National Field Station at a site named Shuirebaozha (Hydrothermal Explosion, 24.95016 °N, 98.43592 °E). This site has shallow features with many geothermal water and gas sources. Samples were collected and their temperature measured in June 2013. Soil column samples were collected by a shock-piston type corer. The maximum depth of the soil was 60 cm, the maximum temperature was 90.8 °C (Table 2), and pH ranged from 8.04 to 8.28. Each 5-cm-depth soil layer was collected as a sample and placed into 1.5 mL or 50 mL polypropylene tubes. All samples for microbial diversity analysis were stored on dry ice during transportation and then at −20 °C in the laboratory until analysis. Samples for microbial culture were stored at 4 °C.

### Cultivation of ammonia-oxidizing microorganisms and activity test

Microbial cells were grown in synthetic media containing NaCl (1 g L^−1^), MgCl_2_·6H_2_O (0.4 g L^−1^), KCl (0.5 g L^−1^), K_2_PO4 (0.75 g L^−1^), NaH_2_PO_4_ (0.25 g L^−1^), MnSO_4_·4H_2_O (0.01 g L^−1^), and CaCO_3_ (5 g L^−1^). After autoclaving, 1 mL ammonium chloride (1 M), 1 mL trace element mixture^41^ and 1 mL vitamin solution^41^ per litre of media were added aseptically. Enrichment cultures were established using allylthiourea (ATU) and chlorate to selectively inhibit the growth of both AOB and nitrite-oxidizing bacteria (NOB). The pH was adjusted to 7.0–7.2. Soil samples (0.1 g) from the different depth layers were inoculated into 10 mL media in glass tubes under dark conditions. The temperature was set according to the environmental temperature. Growth was monitored by microscopy and nitrite production. After cultivation for 24 hours, the ammonia-oxidizing organisms were directly DAPI-stained and then counted on filters using fluorescence microscopy. Ammonium concentration was monitored by Stieglmeier’s^1^ method. Nitrite concentration was determined spectrophotometrically using Griess reagent. Then, the cultures were transferred to slides for morphology determination using scanning electron microscopy (Phenom G2 pro, Phenom-World B.V., Eindhoven, The Netherlands). The reported values are average values from triplicates. The ammonium oxidation rate was measured using the following equation: Ammonium oxidation rate = the increased nitrite concentration/cells number/cultivation days. The increase in nitrite at a given cell density under exponential growth.

### Soil microorganism counts using CARD-FISH

For detection, 0.5 g of soil was used. The preliminary treatment was performed according to a previously reported method^23^. For fixation, 320 μL 25% (w/v) particle free paraformaldehyde solution (4% final concentration) was added, filled up with 1 × PBS, mixed up completely, and the suspension was stored at 4 °C for 24 h. The fixed samples were washed twice with 1 × PBS, centrifuged at 10,000 × g for 5 min at 4 °C after each washing, and stored in PBS/ethanol (1:1) at −20 °C for further processing. Then, 100 μL of the fixed sample was diluted with 900 μL PBS/ethanol and dispersed by ultrasound with an ultrasonic probe at minimum power for 10 s using 1-s sonication pulses. An amount of 20 μL of the dispersed sample was diluted in 10 mL MQ water. This suspension was filtered on polycarbonate filters (0.2 mm pores, 25 mm in diameter), which were mounted in a glass holder for the filtration (If the signal intensity is low, the soil sample dilution rate will be reduced accordingly). After filtration the filters were dipped in 0.1% low melting point agarose and dried in an incubator at 46 °C. The cell walls were permeabilized by addition of proteinase K solution (15 μg/mL, Roche), followed by incubation in 3% H_2_O_2_ to inactivate endogenous peroxidases. Air-dry filters and cut filter sections were used for hybridization. Filter sections were placed in a 1.5-mL tube and mixed with 300 μL hybridization buffer^21^ (0.9 M NaCl, 20 mM Tris–HCl [pH 8.0], 10% (w/v) dextran sulphate, 2% (w/v) blocking reagent (Roche, Germany), 0.1% (w/v) sodium dodecyl sulphate, 55%(v/v) formamide) and 1 μL probe working solution (final concentration 0.028 μM). The probes are shown in Table 1. As a control for unspecific binding, the nonsense probe NONEUB was used.

After hybridization at 46 °C for at least 90 min on a rotor, the filters were transferred to pre-warmed washing buffer^21^ (3 mM NaCl, 5 mM EDTA [pH 8.0], 20 mM Tris–HCl [pH 8.0], 0.01% (w/v) SDS) for 15 min at 48 °C, followed by mixing with 1000 μL of amplification buffer^21^ (1 × PBS [pH7.4], 0.0015% H_2_O_2_, 0.1% (w/v) blocking reagent) and 1 μL of Alexa488 tyramides (molecular probes, Life Technologies^TM^). Then, the filter sections were incubated in amplification buffer at 46 °C for at least 20 min in the dark. Afterwards, the filters were stained by DAPI and mounted with 5 μL droplets of antifade reagent (molecular probes, Life Technologies^TM^). Cell counting was performed on 10 randomly selected micrographs taken with objectives 20 × (150,415 μm^2^) and extrapolated to 1 g of soil. Automated counting was performed with the image analysis software Image J with micrographs showing a high contrast between stained cells and background fluorescence.

### Amplification, cloning and phylogenetic analysis of 16S rDNA and amoA genes

Total DNA was extracted from 500 mg of soil using the FastDNA SPIN Kit for Soil (MP Biomedical, USA) according to the manufacturer’s protocol, with a final elution in 50 μL MQ water. DNA concentration and quality were assessed based on spectral absorbance at 260-nm wavelength and absorbance ratios of 260/280 nm and 260/230 nm, respectively, using a NanoVue Plus Spectrophotometer (GE, USA). DNA was stored at −20 °C until ready for PCR and real-time PCR. PCR amplifications of Archaeal and Bacterial 16S rDNA and amoA genes were performed directly from extracted DNA, using general primers (Table 4). Each 25 μL PCR mixture contained 1.25 U Taq polymerase (Tiangen, Beijing, China), 500 μM dNTP each, 20 mM Tris-HCl (pH 8.3), 100 mM KCl, 3 mM MgCl_2_, 0.4 μM of each primer and PCR-enhancing substances. For 16S rDNA, the following protocol was followed: 95 °C for 5 min; 35 cycles consisting of 94 °C for 60 s, 55 °C for 60 s, and 72 °C for 90 s; and 72 °C for 10 min. For the amoA gene, the following protocol was used: 95 °C for 5 min; 30 cycles consisting of 94 °C for 45 s, 50 °C for 45 s, and 72 °C for 60 s; and 72 °C for 10 min. Triplicate PCR products were pooled (to minimize PCR bias) and gel-purified using the Agarose Gel DNA Fragment Recovery Kit Ver. 2.0 (TaKaRa, Dalian, China). The resulting PCR product quantities were determined by measuring absorbance at 260 nm.

### Construction of 16S rDNA and amoA gene clone libraries

Gel purified PCR products were ligated using the pMD19-T Vector Cloning Kit (TaKaRa, Dalian, China) and transformed into *E. coli* DH5α competent cells according to the manufacturer’s instructions. After transformation, *E. coli* DH5α cells were incubated overnight on LB-agar plates containing 100 μg/mL ampicillin, 40 μg/mL X-gal and 24 μg/mL IPTG at 37 °C. White colonies were selected and PCR-screened for the presence of inserts by using M13F and M13R vector primers. Thirty positive clones per library were randomly selected for sequencing.

### Phylogenetic analysis

The obtained 16S rDNA and amoA gene sequences in each clone library were subjected to operational taxonomic unit (OTU) analysis by MOTHUR, with cutoffs of 3% for 16S rDNA and 2% for amoA gene sequences. The potential presence of chimeric sequences was examined with Bellerophon (http://comp-bio.anu.edu.au/bellerophon/bellerophon.pl).

One sequence from each OTU was selected as a representative, and the closest reference sequences (GenBank: http://www.ncbi.nlm.nih.gov & RDP) were pooled and aligned using CLUSTAL X. Phylogenetic analysis was performed using the distance-based maximum likelihood method with MEGA version 6.0. Bootstrap analysis was performed using 1000 replications. The diversity indices of Shannon-Weaver (H’) and Chao1 were also calculated using MOTHUR.

### Real-time PCR

The 16S rDNA and amoA gene copies of Bacteria and Archaea were quantified by real-time PCR with SYBR Green. Each reaction was performed in a 20-μL volume containing 10 ng soil DNA, 0.3 μM of each primer and 10 μL of SupelReal PreMix Plus SYBR Green (Tiangen, Beijing, China). The real-time PCR protocol and primers were performed as described above. The initial denaturation was carried out at 95 °C for 15 min with 40 amplification cycles. Reactions were carried out in an ABI Prism 7500 real-time PCR system. PCR product was confirmed by melting curve analysis and visualization with agarose gels showing specific product bands of the expected size. An external standard curve for 16S rDNA and the amoA gene was generated (Fig. S2). The PCR product was cloned with a pMD™19-T Vector Cloning Kit (TaKaRa, China). The plasmid concentration was measured with a spectrophotometer. The copy numbers of genes were calculated directly from the concentration of extracted plasmid DNA. For Bacterial and Archaeal 16S rDNA gene quantification, the standard curve contained 20 to 2.21 × 10^9^ copies of template per assay; for quantification of AOB and AOA amoA, the templates copy number varied between 10 and 10^8^ copies per assay, with correlation coefficient R^2^ values between 0.9816 and 0.9998 and slopes between −3.7078 and −3.6913. Water was used instead of DNA as a non-template control.

### Nucleotide sequence accession numbers

The sequence data from this study have been deposited in GenBank under accession numbers KM585413-KM585536.

## Additional Information

**How to cite this article**: Li, H. *et al.* The impact of temperature on microbial diversity and AOA activity in the Tengchong Geothermal Field, China. *Sci. Rep.* **5**, 17056; doi: 10.1038/srep17056 (2015).

## Supplementary Material

Supplementary Information

## Figures and Tables

**Table 1:** Oligonucleotide probes used in CARD-FISH experiments.

Probe Name	Target	Sequence (5′–3′)
EUB338 (I–III)	Bacteria	Mixture of the probes EUB338, EUB338 II, and EUB338 III
EUB338 I	Most Bacteria	GCT GCC TCC CGT AGG AGT
EUB338 II	Planctomycetes	GCA GCC ACC CGT AGG TGT
EUB338 III	*Verrucomicrobium*	GCT GCC ACC CGT AGG TGT
NONEUB	Nonsense of EUB338	ACT CCT ACG GGA GGC AGC
Arch915	Archaea	GTG CTC CCC CGC CAA TTC CT
Cren512	most Crenarchaeota	CGG CGG CTG ACA CCA G

**Table 2:** Abundance of 16S rRNA and amoA genes and cell number information for different soil samples collected from Tengchong.

Sample site	Soil depth & layer (cm)	T ( °C)	CARD-FISH (cells g^−1^ soil)				Gene copies (g^−1^ soil)		
			Bacterial (SD)	Archaea (SD)	AOA (SD)	DAPI (SD)	Bacterial (SD)	Archaea (SD)	AOA (SD)
Up stream	0–10	52.3	6.0 × 10^7^	7.2 × 10^6^	2.0 × 10^2^	7.3 × 10^7^	8.78 × 10^7^	8.20 × 10^6^	5.34 × 10^2^
	10–20	64.3	7.0 × 10^6^	5.5 × 10^5^	2.0 × 10^2^	9.5 × 10^6^	9.83 × 10^6^	7.00 × 10^5^	1.28 × 10^2^
	20–30	74.6	3.5 × 10^4^	4.5 × 10^4^	3.0 × 10^2^	1.5 × 10^5^	3.29 × 10^4^	5.92 × 10^4^	1.64 × 10^2^
Middle stream	0–10	64.2	6.6 × 10^6^	4.7 × 10^5^	1.9 × 10^2^	7.3 × 10^6^	8.37 × 10^6^	4.53 × 10^5^	1.25 × 10^2^
	10–20	80.0	4.2 × 10^3^	3.5 × 10^4^	4.0 × 10^3^	5.5 × 10^4^	5.50 × 10^3^	2.53 × 10^4^	5.28 × 10^3^
	20–30	89.9	5.5 × 10^2^	4.5 × 10^3^	1.0 × 10^3^	5.3 × 10^3^	1.60 × 10^2^	6.21 × 10^3^	1.06 × 10^3^
	30–40	90.8	0.5 × 10^2^	3.6 × 10^3^	3.0 × 10^3^	4.0 × 10^3^	1.17 × 10^2^	3.89 × 10^3^	3.30 × 10^3^
Down stream	0–10	66.5	6.4 × 10^6^	4.3 × 10^5^	1.0 × 10^2^	7.8 × 10^6^	8.23 × 10^6^	5.08 × 10^5^	1.21 × 10^2^
	10–20	71.3	8.9 × 10^5^	4.0 × 10^5^	5.0 × 10^2^	3.5 × 10^6^	7.76 × 10^5^	4.08 × 10^5^	5.44 × 10^2^
	20–30	76.6	3.5 × 10^4^	5.3 × 10^4^	1.0 × 10^3^	1.3 × 10^5^	3.24 × 10^4^	5.64 × 10^4^	1.23 × 10^3^
	30–40	79.3	4.5 × 10^3^	4.0 × 10^4^	1.1 × 10^3^	5.1 × 10^4^	8.51 × 10^3^	4.98 × 10^4^	1.39 × 10^3^

**Table 3:** Shannon–Weaver “H”) and Chao1 indices for each sample 16S rRNA gene clone library along with the number of OTUs “S”, evenness “E” and geochemistry conditions.

Sample		Index for clone library				Geochemistry mg/L		
		S	E	Chao1	H	NH_4_^+^	NO_2_^−^	NO_3_^−^
52.3 °C	Bacteria	16	0.59	21	2.37	3.9	0.28	3
	Archaea	11	0.53	12	1.82			
	AOA	6	0.46	6	1.18			
64.3 °C	Bacteria	14	0.56	16	2.13	3.8	0.20	2
	Archaea	10	0.56	11	1.86			
	AOA	6	0.51	6.6	1.15			
74.6 °C	Bacteria	10	0.56	11	1.86	2.36	0.12	1.2
	Archaea	7	0.48	7.5	1.35			
	AOA	5	0.44	5	1.02			
80.0 °C	Bacteria	5	0.44	5	1.02	1.84	0.12	1.9
	Archaea	7	0.45	7.3	1.32			
	AOA	4	0.47	4	0.95			
90.8 °C	Bacteria	3	0.51	3	0.81	1.89	0.20	2.5
	Archaea	4	0.47	4	0.95			
	AOA	3	0.47	3	0.81			

**Table 4:** Primers used for PCR amplifications of archaeal and bacterial 16S rDNA and AOA amoA genes.

Target	Primer	Annealing temperature	Sequence (5′–3′)
Bacteria	Bac27F	55 °C	AGA GTT TGA TCN TGG CTC AG
	Bac1492R		TAC GGY TAC CTT GTT ACG ACT T
Archaea	Arch21F	55 °C	TTC CGG TTG ATC CYG CCG GA
	Arch958R		YCC GGC GTT GAM TCC AAT T
AOA	Arch amoAF	50 °C	AAT GGT CTG GCT WAG ACG C
	Arch amoAR		GAC CAR GCG GCC ATC CA
