# Supplementary material for: The impact of temperature on microbial diversity and AOA activity in the Tengchong Geothermal Field, China
Source: Sci Rep. 2015 Nov 26;5:17056. doi: 10.1038/srep17056 (PMC4660298; doi:10.1038/srep17056)
Supplement: Supplementary Information [file srep17056-s1.doc]

**The impact of temperature on microbial diversity and AOA activity in the Tengchong** **Geothermal Field, China**

**Haizhou Li^1^, Qunhui Yang****^2^, Jian Li^3^, Hang Gao^2^, Ping Li^1^*** **& Huaiyang Zhou^2^***

Correspondence and requests for materials should be addressed to Huaiyang Zhou, zhouhy@tongji.edu.cn, or Ping Li liping01@tongji.edu.cn

1: School of Life Sciences and Technology, Tongji University, Shanghai 200092, China;

2: State Key Laboratory of Marine Geology, School of Ocean and Earth Science, Tongji University, Shanghai 200092, China;


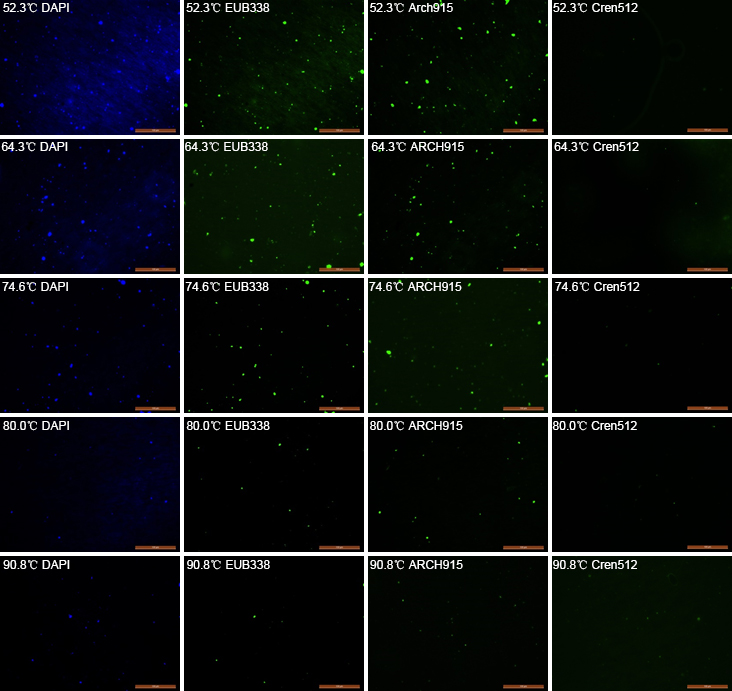
3: School of Engineering, Anhui Agricultural University, Hefei 230000, China.

**Figure S1** Photomicrographs of CARD-FISH stained samples.

DAPI stained in blue. Bacterial probes (EUB338 I–III), Archaea probes (ARCH915) , Crenarchaeota probe(Cren512). Magnification=200×, scale bar=100μm

A


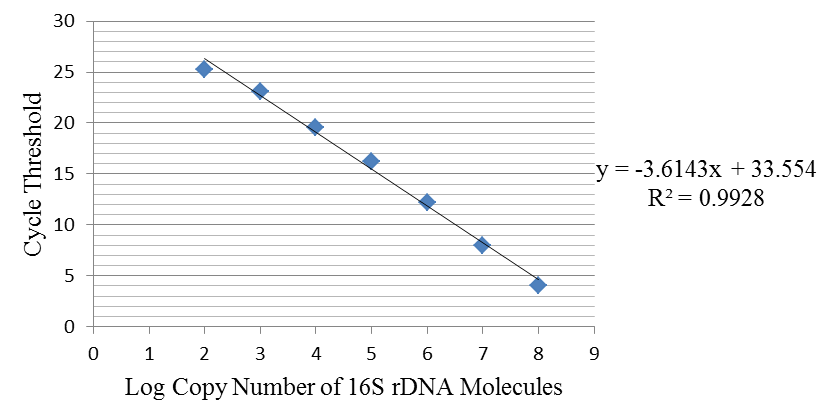


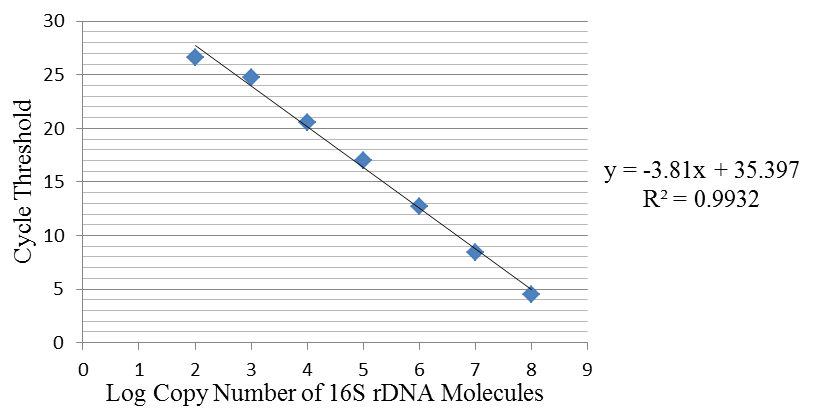
B


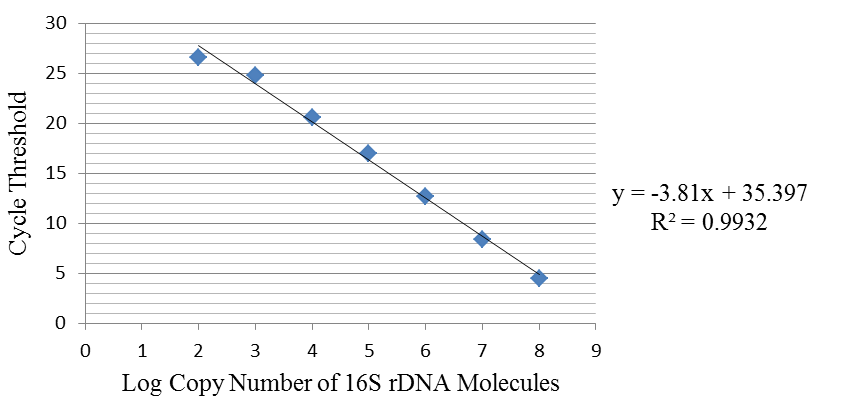
C

**Figure S2** An external standard curve for Real-time PCR. (A) Standard curves for quantification of Archaeal 16S rDNA. (B) Bacterial 16S rDNA genes. (C) AOA amoA genes. DNA was used in seven 10^-1^ dilution steps.

**Table S1** Summary of microorganism’s OTUs detected in 16S rRNA gene clone libraries. For each OTU, the closest GenBank match (based on RDP-II SEQMATCH and BLAST search tool) with accession number and percentage sequence similarity, class-level affiliation are given.

| Closest GenBank match (% identity, Accession No.) | The classification of microorganisms | Sample source | Optima of temperature |
| --- | --- | --- | --- |
| *Caldisericum exile* gen. nov.,  90~97% NC_017096.1 | Bacteria;  Caldiserica;  Caldisericia;  Caldisericales;  Caldisericaceae;  *Caldisericum* | 74.6  80.0  90.8 | 75℃ |
| *Desulfomonile tiedjei* DSM 679995~97%NC_018025.1 | [Bacteria](http://www.ncbi.nlm.nih.gov/Taxonomy/Browser/wwwtax.cgi?mode=Undef&id=2&lvl=3&keep=1&srchmode=1&unlock);  [Proteobacteria](http://www.ncbi.nlm.nih.gov/Taxonomy/Browser/wwwtax.cgi?mode=Undef&id=1224&lvl=3&keep=1&srchmode=1&unlock);  [delta/epsilonsubdivisions](http://www.ncbi.nlm.nih.gov/Taxonomy/Browser/wwwtax.cgi?mode=Undef&id=68525&lvl=3&keep=1&srchmode=1&unlock);  [*Deltaproteobacteria*](http://www.ncbi.nlm.nih.gov/Taxonomy/Browser/wwwtax.cgi?mode=Undef&id=28221&lvl=3&keep=1&srchmode=1&unlock) | 50.3 | 40℃ |
| *Thauera* sp. MZ1T 91~98%NC_011662.2 | Bacteria;  Proteobacteria; Betaproteobacteria; Rhodocyclales;  Rhodocyclaceae;  *Thauera* | 50.3 | 40℃ |
| *Rubrivivax gelatinosus* IL144  95~98% NC_017075.1 | Bacteria;  Proteobacteria; Betaproteobacteria; Burkholderiales;  *Rubrivivax* | 50.3 | 37℃ |
| *Methylococcus capsulatus* 97~100%NC_002977.6 | Bacteria;  Proteobacteria; Gammaproteobacteria;  Methylococcales;  Methylococcaceae*; Methylococcus* | 50.3 | 40℃ |
| *Haemophilus parainfluenzae* 97~100*%*NC_015964.1 | Bacteria;  Proteobacteria; Gammaproteobacteria; Pasteurellales;  Pasteurellaceae;  *Haemophilus.* | 50.3 | 40℃ |
| *Methylobacterium extorquens* DM4  97~100%NC_012988.1 | Bacteria;  Proteobacteria; Alphaproteobacteria;  Rhizobiales;  Methylobacteriaceae; *Methylobacterium* | 50.3 | 37℃ |
| *Novosphingobium aromaticivorans* DSM 12444  98% NC_007794.1 | Bacteria;  Proteobacteria; Alphaproteobacteria; Sphingomonadales;  Sphingomonadaceae; *Novosphingobium* | 50.3 | 37℃ |
| *Syntrophus aciditrophicus* SB  89~98%NC_007759.1 | Bacteria;  Proteobacteria; Deltaproteobacteria; Syntrophobacterales;  Syntrophaceae;  *Syntrophus* | 90.8 | 76℃ |
| *Geobacter uraniireducens Rf4*  84~95% NC_009483.1 | Bacteria;  Proteobacteria;  Deltaproteobacteria;  Desulfuromonadales;  Geobacteraceae;  *Geobacter* | 90.8 | 88℃ |
| *Hippea maritima DSM 10411*  80-~94%NC_015318.1 | Bacteria;  Proteobacteria; Deltaproteobacteria; Desulfurellales;  Desulfurellaceae;  *Hippea* | 80.2  90.8 | 74℃ |
| *Chloroflexus aggregans* DSM 9485  97~100%NC_011831.1 | Bacteria;  Chloroflexi;  Chloroflexia;  Chloroflexales;  Chloroflexineae; Chloroflexaceae;  *Chloroflexus* | 52.3  64.3 74.6 | 70℃ |
| *Anaerolinea thermophila* 85~97% NC_014960.1 | Bacteria;  Chloroflexi;  Anaerolineae;  Anaerolineales;  Anaerolineaceae;  *Anaerolinea* | 52.3  64.3 74.6 | 70℃ |
| *Dehalococcoides* sp. GT 84~95%NC_013890.1 | Bacteria;  Chloroflexi;  Dehalococcoidia; Dehalococcoidales;  Dehalococcoidaceae; *Dehalococcoides.* | 52.3  64.3 74.6 | 72℃ |
| *Thermodesulfatator indicus* DSM 15286 97~100% NC_015681.1 | Bacteria;  Thermodesulfobacteria; Thermodesulfobacteriales;  Thermodesulfobacteriaceae;  *Thermodesulfatator* | 74.6  80.2  92.3 | 74℃ |
| *Thermotoga petrophila* RKU-182% NC_009486.1 | Bacteria;  Thermotogae;  Thermotogales;  Thermotogaceae;  *Thermotoga.* | 80.2  92.3 | 85℃ |
| [*Thermodesulfovibrio yellowstonii* DSM 11347](http://www.ncbi.nlm.nih.gov/Taxonomy/Browser/wwwtax.cgi?id=289376)  89~79%NC_011296.1 | Bacteria;  Nitrospirae;  Nitrospirales;  Nitrospiraceae*;*  *Thermodesulfovibrio* | 74.6  80.2 | 70℃ |
| [*Desulfomonile tiedjei* DSM 6799](http://www.ncbi.nlm.nih.gov/Taxonomy/Browser/wwwtax.cgi?id=706587)  97~100%NC_018025.1 | Bacteria;  Caldiserica;  Caldisericia;  Caldisericales;  Caldisericaceae;  *Caldisericum* | 64.3  74.6 | 65℃ |
| *Ammonifex degensii* KC4 94~98%NC_013385.1 | Bacteria;  Firmicutes;  Clostridia; Thermoanaerobacterales;  Thermoanaerobacteraceae; Moorella group;  *Ammonifex* | 74.6  80.2  92.3 | 75℃ |
| *Caldanaerobacter uzonensis strain K67*  95~98% EF195126.1 | Bacteria;  Firmicutes;  Clostridia; Thermoanaerobacterales;  Thermoanaerobacteraceae;  *Caldanaerobacter* | 64.3  74.6  80.2 | 75℃ |
| *Ignavibacterium album* JCM 16511  95~98%NC_017464.1 | Bacteria;  Ignavibacteriae;  Ignavibacteria;  Ignavibacteriales;  Ignavibacteriaceae; *Ignavibacterium* | 74.6  80.2  90.3 | 72℃ |
| *Cyanothece* sp. PCC 8802  75~96%NC_013161.1 | Bacteria;  Cyanobacteria; Oscillatoriophycideae; Chroococcales;  *Cyanothece* | 50.3 | 37℃ |
| *The**rmogladius cellulolyticus* 1633  92~98% NC_017954.1 | Archaea;  Crenarchaeota;  Thermoprotei;  Desulfurococcales;  Desulfurococcaceae; *Thermogladius.* | 50.3  64.3  74.6 | 74 °C |
| *Hyperthermus butylicus* DSM 5456  92~98NC_008818.1 | Archaea;  Crenarchaeota;  Thermoprotei;  Desulfurococcales;  Pyrodictiaceae;  *Hyperthermus* | 90.8 | 95℃ |
| *Vulcanisaeta distributa* DSM 14429  94~97%NC_014537.1 | Archaea;  Crenarchaeota;  Thermoprotei;  Thermoproteales;  Thermoproteaceae;  *Vulcanisaeta* | 80.2  90.8 | 89℃ |
| *Thermofilum* sp. 1910b 97~98%CP006646.1 | Archaea;  Crenarchaeota;  Thermoprotei;  Thermoproteales;  Thermofilaceae;  *Thermofilum.* | 80.2  90.8 | 85℃ |
| Methanocaldococcus sp. FS406-22 NC_013887.1 | Archaea;  Euryarchaeota;  Methanococci;  Methanococcales; Methanocaldococcaceae; *Methanocaldococcus* | 80.2  90.8 | 80℃ |
| *Methanosaeta thermophila* strain PT  95~98%NR_074214.1 | Archaea;  Euryarchaeota;  Methanomicrobia; Methanosarcinales; Methanosaetaceae;  *Methanosaeta* | 74.6  80.2  90.8 | 82℃ |
